# Efficacy of Tranexamic Acid in a Large Population with Proximal Femur Fractures—A Matched Pair Study of 7394 Patients from the Registry for Geriatric Trauma (ATR-DGU)

**DOI:** 10.3390/jcm15187147

**Published:** 2026-09-15

**Authors:** Ulf Bökeler, Ulrich Liener, Hannah Schmidt, Daphne Eschbach, Rene Aigner, Steffen Ruchholtz, Tom Knauf, on behalf of the Registry for Geriatric Trauma (ATR-DGU)

**Affiliations:** 1 Department for Orthopaedics and Trauma Surgery, Marienhospital Stuttgart, Böheimstrasse 37, 70199 Stuttgart, Germany; 2 AUC-Academy for Trauma Surgery, 80538 Munich, Germany; 3 Department of Orthopedic and Trauma Surgery, Helios Kliniken Kassel, 34121 Kassel, Germany; 4 Center for Orthopaedics and Trauma Surgery, University Hospital Giessen and Marburg, 35043 Marburg, Germany

**Keywords:** hip fracture, fragility fracture, blood loss, tranexamic acid, registry

## Abstract

**Background:** Perioperative blood loss represents a major challenge in elderly patients undergoing hip fracture surgery. Although tranexamic acid (TXA) has been shown to reduce bleeding complications, evidence from large registry-based cohorts remains limited. This study assessed the efficacy and safety of perioperative TXA administration in geriatric proximal femoral fracture surgery and evaluated in a subgroup analysis whether repeated TXA administration provides additional benefit compared with a single intraoperative dose. **Methods:** Data were derived from the Registry for Geriatric Trauma of the German Trauma Society (ATR-DGU), a prospective multicenter registry of patients aged ≥70 years undergoing surgery for proximal femoral fractures. From a total cohort of 23,830 patients, 1:1 nearest-neighbor matching based on age, sex, America Society of Anesthesiologists (ASA) classification, fracture type, and pre-fracture mobility yielded 3697 patients receiving intraoperative TXA and 3697 controls without TXA. Outcomes included transfusion requirements, thromboembolic events, mortality, reoperation rate, and length of stay. A subgroup analysis compared single-dose intraoperative TXA with combined pre- and intraoperative administration. **Results:** Intraoperative TXA significantly reduced packed red blood cell transfusion rates compared with controls (44.6% vs. 52.2%; *p* < 0.001). No significant differences were observed in thromboembolic events, mortality, overall complication rates, or length of hospital stay. Subgroup analysis showed no additional benefit of repeated pre- and intraoperative TXA administration compared with a single intraoperative dose but was associated with a significantly higher incidence of thromboembolic risks in the multiple-dose cohort (3.56% vs. 1.1%; *p* = 0.038). **Conclusions:** Intraoperative TXA administration significantly reduces transfusion requirements in geriatric patients undergoing surgery for proximal femoral fractures without increasing thromboembolic complications or mortality. TXA should therefore be considered a safe and effective adjunct in the perioperative management of geriatric hip fracture patients.

## 1. Introduction

The global number of hip fractures is projected to be increased by 2050 to 6.25 million [1]. Hip fractures can lead to decreased quality of life, disability and increased mortality [2]. Elderly patients face a high risk of dying after a hip fracture; the in-house mortality rate ranges between 5 and 7%. Up to 30% of these patients die within the first year after fracture [3,4,5]. Intracapsular (cervical) and extracapsular (trochanteric and subtrochanteric) fractures are the two main fracture types. Apart from a few exceptions, early surgery is essential [6]. Depending on biological factors and the fracture morphology, total or hemi arthroplasty, intramedullary nailing or dynamic hip screw fixation are performed. The amount of blood loss is crucial in these vulnerable, geriatric patients, and substantial perioperative blood loss and subsequent anemia or hypovolemia may trigger a clinical deterioration of cardiac and renal functions [7,8]. Post-operative Anemia Necessitating Transfusion (PANT) is associated with an increased time to mobilization, increased length of stay, higher infections and incidences of venous thromboembolism, and an increasing risk of allergic reactions [9,10,11]. Numerous studies have shown that tranexamic acid administered intravenously during surgery reduces blood loss and lowers transfusion rates without raising the risk of complications in hip and knee arthroplasty [12,13] and hip fracture surgery [14,15,16,17,18,19]. Tranexamic acid (TXA) is an antifibrinolytic agent that competitively binds to the lysine-binding sites of plasminogen. It prevents the binding of fibrin to the plasminogen-plasmin tissue activator complex and therefore blocks the degradation of fibrin [20]. Despite the relatively large number of studies investigating the efficacy of tranexamic acid in the surgical management of proximal femoral fractures, large-scale trials involving substantial patient cohorts remain scarce [14,15,16,17,18,19]. Furthermore, the frequency of tranexamic acid administration remains unclear. In the setting of primary TKA, giving a preoperative and intraoperative dose of intravenous (IV) TXA has demonstrated superior blood-sparing properties compared with a single dose of IV TXA [21,22].

The objective of this study was to evaluate the efficacy and safety of TXA therapy within an extensive patient cohort. Furthermore, a subgroup analysis was conducted to determine whether additional administrations preoperatively, compared to a single dose administered immediately prior to surgery, result in any significant differences in the complication rate and the postoperative transfusion rate.

## 2. Methods

### 2.1. Source of Data

The data for this analysis were derived from the Registry for Geriatric Trauma (ATR-DGU) [23], a prospective, multicenter, and standardized database established by the German Trauma Society (DGU) in 2016. The ATR-DGU captures comprehensive clinical data on patients aged 70 years and older who have sustained hip, periprosthetic, or peri-implant femoral fractures requiring surgical intervention.

The scientific oversight is provided by the Working Committee on Geriatric Trauma Registry (AK ATR) of the DGU, while the AUC—Academy for Trauma Surgery—manages the technical infrastructure, including data management and analysis. Participating centers—primarily located in Germany, Switzerland, and Austria—submit pseudonymized patient data via a secure, web-based platform. To date, the registry encompasses approximately 100,000 cases from 196 certified hospitals.

Hospitals contributing to the registry must achieve certification as an “Centre for Geriatric Trauma” (AltersTraumaZentrum DGU^®^, ATZ) (ATZ-DGU). This certification necessitates adherence to rigorous interdisciplinary standards, including:

- Collaborative treatment by orthopedic surgeons and geriatricians.
- Implementation of Standard Operating Procedures (SOPs) for surgical care and pain management.
- Structured protocols for mobilization, delirium screening, osteoporosis assessment, and discharge planning.

Data acquisition follows a longitudinal approach across five distinct clinical phases: admission, preoperative, intraoperative, the first postoperative week, and discharge, with an optional follow-up at 120 days.

The present study (Project ID: 2023-002) complies with the ATR-DGU publication guidelines. Access to the data was granted following a formal peer-review process conducted by the AK ATR.

### 2.2. Patients

This study exclusively utilized data from the years 2022 and 2023 based on the 2022 registry version, as this iteration introduced the collection of specific data regarding the pre- and perioperative administration of tranexamic acid. The total cohort utilizing the 2022 documentation version comprises 32,934 cases across 163 hospitals. Valid data regarding the pre- and intraoperative administration of tranexamic acid were available for 27,978 of these cases.

### 2.3. Patient Matching

To control for differences between the demographics in patients who received tranexamic acid intraoperatively and those who did not, a 1:1 nearest-neighbor matching was conducted. Sex, ASA score, fracture type, age and walking ability before the fracture were considered as matching variables. Matching was performed using the Matchlt pachage (Version 4.5.5., Ho, Imai, King&Stuart, 2011). Following matching, all covariates exhibited an absolute standardized mean difference (ASMD) below 0.02, demonstrating a high degree of balance and ensuring comparability between the treatment groups.

### 2.4. Exposure

Outcome was measured by the following parameters: postoperative non-operative complications, including thrombosis, pulmonary embolism and myocardial infarction, reoperation rate, length of hospital stay (LOS), and requirement for hemostatic therapy (administration of packed red blood cells, fresh frozen plasma, and platelet concentrate).

### 2.5. Statistics

All statistical analyses were performed using R statistical software (version 4.3.2, Foundation for Statistical Computing, Vienna, Austria). The baseline characteristics and outcome variables of the study population are provided using descriptive statistics. The data are presented as mean value, standard deviation, minimum and maximum for parametric continuous variables, medians with interquartile ranges (IQRs) for non-parametric continuous variables or counts and percentages for categorical variables. Due to missing data for certain parameters, the analyses reflect the number of patients included in each specific assessment.

Group comparisons (TXA versus No TXA) were conducted using Student’s *t*-test or the Wilcoxon test for continuous data, as appropriate, and the Pearson chi-squared test for categorical data. Fisher’s exact test was applied for categorical variables with fewer than five expected observations. The *p*-values of these comparisons should be interpreted with caution, as in large sample sizes even small differences can be significant. Conditional logistic regression was used to estimate odds ratios (ORs) and 95% confidence intervals (CIs), with matched pairs treated as strata.

The subgroup analysis (2xTXA versus TXA) was conducted in the same way, but due to the smaller sample size, a 2:1 matching was performed.

### 2.6. Ethics

All patients consented in writing to the study. Written consent from all patients is a prerequisite to participate in the ATR-DGU. Without this, entry into the register is not possible. A positive evaluation of the ethics committee of the University of Marburg was obtained (protocol code AZ/46/16 and 4 May 2018 of approval).

## 3. Results

**Figure 1:**
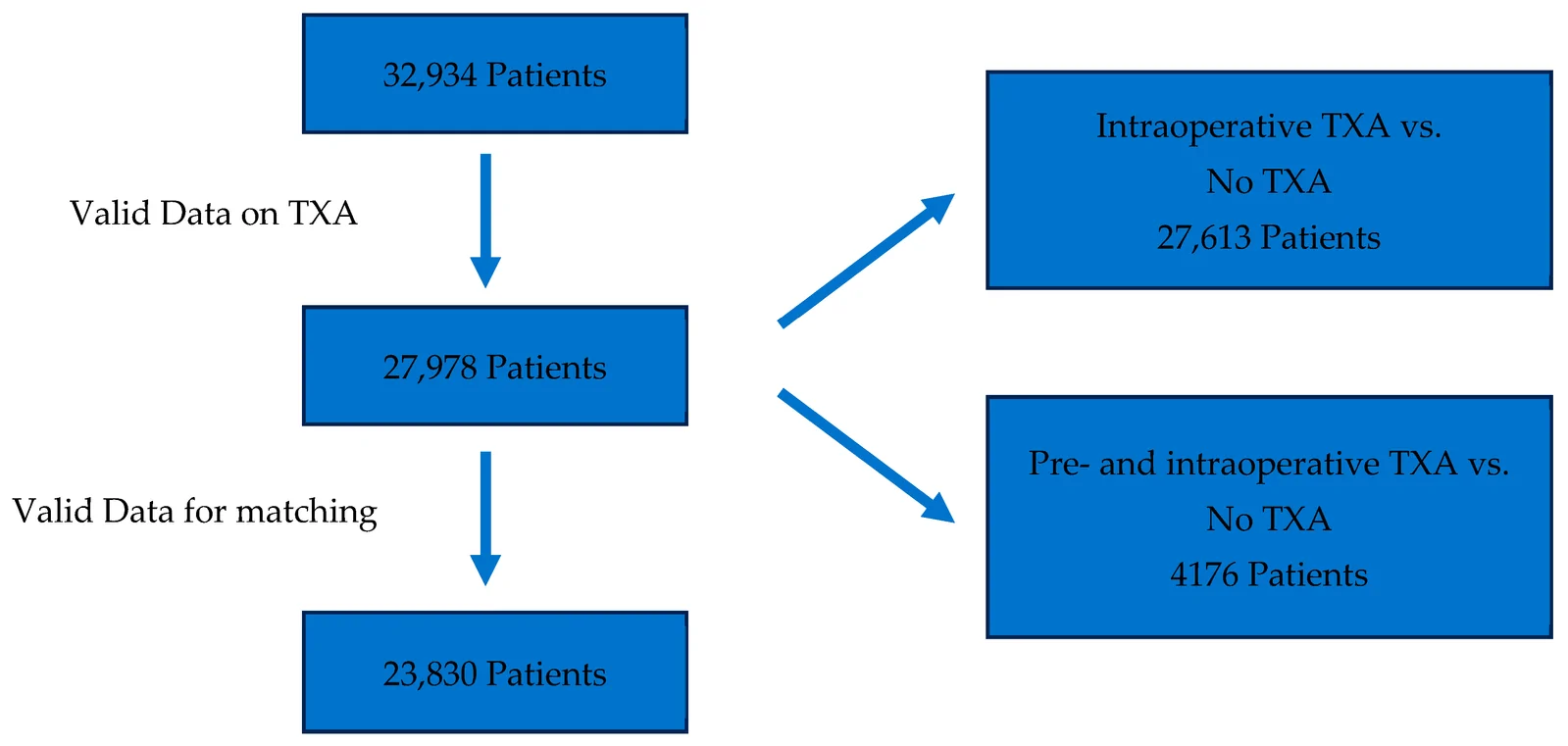
TXA—Tranexamic acid.

Between 2022 and 2023, a total of 32,934 patients were entered into the register. Valid data on the pre- and intraoperative administration of TXA were available for 27,978 of these patients. For the matching process, patients were grouped according to age, ASA classification, gender, walking ability prior to the fracture, and fracture type. For 23,830 patients, complete data were available. Further information is shown in Figure 1.

After the nearest-neighbor matching method, finally, 3697 patients receiving intraoperative TXA and 3697 patients in the control group (No TXA) were compared with each other.

After matching, patients in the intraoperative TXA group and patients who received no TXA showed marginal differences in age, sex, fracture type, ASA and walking ability. Further pre-surgery data are shown in Table 1.

At admission, 52.8% (*n* = 1916) of the TXA group patients were on anticoagulants, compared to 50.31% (*n* = 1797) in the group not receiving tranexamic acid (*p* = 0.037). A detailed breakdown of the status is provided in Table 2.

In both groups, additional intraoperative hemostatic therapy, apart from the administration of tranexamic acid, was rarely performed. In the tranexamic acid group, the intraoperative administration of fibrinogen, prothrombin complex concentrate (PCC) and reversal agents for non-vitamin K antagonist oral anticoagulants (NOACs) were significantly higher compared to the control group. Regarding the administration of vitamin K, no significant differences were observed between the groups (Table 3).

In both groups the two most common operational procedures were the implantation of a hip prosthesis (bipolar 46.4% (*n* = 1713) in the TXA group and 56.5% (*n* = 2087) in the No TXA group and total hip arthroplasty 23.2% (*n* = 855) in the TXA group vs. 8.0% (*n* = 295) in the No TXA group) and the stabilization with a trochanteric nail (18.6% (*n* = 686), TXA vs. 21.0 (*n* = 777), no TXA). Other surgical procedures like Dynamic Hip Screw, canulated screws and others were rare (4.7% (*n* = 164), TXA vs. 9.3% (*n* = 244), No TXA). The differences are due to the large sample size being statistically significant (*p* < 0.05).

### 3.1. Mortality

The in-hospital mortality was comparable between both groups, 5.34% (TXA) vs. 5.09% (No TXA). Furthermore, there was no significant difference in 120-day mortality between both groups (9.57% vs. 9.69%).

### 3.2. Complications and Reoperation Rate

No significant differences were observed between the two groups regarding non-surgical complications during the inpatient stay, 39.96% (*n* = 1447) in the TXA group vs. 38.84% (*n* = 1401) in the No TXA group. Similarly, thromboembolic events—including myocardial infarction, deep vein thrombosis, and pulmonary embolism—showed no significant variations between the groups, 1.70% (*n* = 63) in the TXA group vs. 1.87% (*n* = 69) in the No TXA group. Furthermore, the 120-day follow-up revealed no statistically significant intergroup differences in either non-surgical complications, 10.42% (*n* = 92) vs. 7.99% (*n* = 64), and the occurrence of thromboembolic events, including myocardial infarction, thrombosis, or pulmonary embolism, 0.39% (*n* = 4) vs. 0.96 (*n* = 9).

The reoperation rate during the inpatient stay was significantly higher in the TXA group compared to the No TXA group, 4.31% (*n* = 156) vs. 3.04 (*n* = 109) (*p* = 0.005). The most common surgical revisions were repositioning following dislocation of the prosthesis (TXA 16.2% vs. No TXA 8.9%), tissue irrigation/debridement (TXA 40.3% vs. No TXA 35.6%) and replacement of a prosthesis or implants (TXA 9.1% vs. No TXA 16.8%).

### 3.3. Hemostatic Management

Intraoperative administration of TXA was associated with a significantly lower requirement for packed red blood cell (pRBC) transfusions (at least one unit) during the inpatient stay, 44.64% (*n* = 996) vs. 52.16% (978), *p* < 0.001. The TXA group received a significantly higher volume of at least one unit for fresh frozen plasma (FFP) compared to the control group (4.66 (*n* = 81) vs. 2.63% (*n* = 35) (*p* = 0.005)); in contrast, there were no significant differences regarding the administration of platelet concentrates between the two groups (1.47% (*n* = 25) vs. 1.13% (*n* = 15)).

### 3.4. Length of Stay, Discharge and Living Situation Postoperative

The median length of stay of patients did not differ significantly between both groups, 15.08 days [9.04, 21.04] vs. 15.04 days [9.04, 21.08]. While a slightly higher proportion of patients in the TXA group were discharged to their homes or long-term care facilities, a greater number of patients in the control group were transitioned to acute geriatric rehabilitation units.

Clinical outcomes—including mortality, complication, and reoperation rates—as well as coagulation management data are summarized in Table 4 and Table 5.

### 3.5. Subgroup Analysis

For the subgroup analysis, complete documentation of matching parameters was available for 3697 cases. Following the nearest-neighbor matching, a total of 675 cases were included: 450 patients in the intraoperative tranexamic acid group (TXA group) and 225 patients in the combined pre- and intraoperative tranexamic acid group (2xTXA group). After matching, there were no significant differences between the patients in the two comparison groups regarding age, ASA classification, sex, walking ability, fracture type and surgical procedure. Furthermore, no significant differences were observed with respect to anticoagulation therapy at the time of hospital admission (see Table 6). Patients who received preoperative and intraoperative tranexamic acid required preoperative treatment with fibrinogen significantly more often (2.4%, *n* = 5) than patients who received tranexamic acid intraoperatively only (0%). Regarding the preoperative and intraoperative administration of PCC and vitamin K, no significant differences were observed between the two groups.

### 3.6. Results of the Subgroup Analysis

With regard to non-surgical complications, the reoperation rate and the lethality during the inpatient stay and in the 120-day follow-up, no significant differences were observed between the groups. There were no significant differences between the two groups regarding the proportionate number of administered blood transfusions, fresh frozen plasma (FFP), and platelet concentrates. A detailed breakdown of these results is provided in Table 7.

## 4. Discussion

Widespread clinical adoption of tranexamic acid as a routine medication for proximal femur fracture surgery remains inconsistent across healthcare settings. In our study, perioperative tranexamic acid was administered to only 15.1% of the total cohort (4176 of 27,613 patients) across the 163 participating hospitals. This observation is particularly noteworthy, as it has been demonstrated that occult blood loss in these vulnerable geriatric patients can lead to a range of well-documented complications [24], and evidence suggests that tranexamic acid effectively mitigates occult blood loss in the surgical treatment of proximal femur fractures [15,17,18,25]. Routine implementation of tranexamic acid protocols in the surgical therapy of geriatric proximal femur fractures often faces skepticism. This hesitance is largely rooted in the select non-orthopedic literature that has reported heterogeneous outcomes for tranexamic acid administration. In cardiac surgery, the ATACAS trial—encompassing 4631 patients—demonstrated that while tranexamic acid (TXA) effectively reduced bleeding, it was associated with a significantly increased risk of seizures. This risk was particularly pronounced in patients undergoing open-chamber cardiac procedures [26].

To our knowledge, this study provides the most robust registry evidence thus far regarding TXA utilization in hip fracture surgery. The data reveal a substantial and statistically significant decrease in transfusion rates following a one-time intraoperative application. Notably, these results underscore the safety of the drug, as no significant correlation was found between TXA use and a higher incidence of thromboembolic events.

Our results are consistent with the current literature on this topic. Fenwick et al. analyzed 1479 patients at a Level I trauma center and observed a 17.1% reduction in transfusion rates without an increase in complication rates [18]. Similarly, a multicenter cohort study by Bai et al. involving 1228 patients aged 65 and older with intertrochanteric fractures demonstrated that all tranexamic acid (TXA) regimens reduced blood loss; notably, the combination of intravenous and topical administration proved most effective [27]. Furthermore, two large-scale meta-analyses consistently show a reduction in transfusion risk of approximately 44–45% with no associated increase in thromboembolic risk [28,29]. The largest prospective registry-based cohort study to date, conducted by Viberg et al., included 3097 patients aged 65 and older with hip fractures [19]. Their findings revealed a significant reduction in the transfusion rate, which decreased from 31% to 27%. Remarkably, their data indicated that TXA was correlated with a reduced incidence of thromboembolic complications, further supporting its safety profile in this vulnerable population. These findings were not replicated in our study. The unexpected outcome that the administration of tranexamic acid (TXA) may reduce the risk of thromboembolic complications was documented in two further studies [30,31]. This potential benefit could be attributed, on the one hand, to a decreased requirement for blood transfusions and, on the other, to the potential intrinsic anti-inflammatory effects of tranexamic acid itself [32]. The thrombogenic potential of blood transfusions is attributed to several mechanisms, including the activation of the coagulation cascade mediated by microparticles and free hemoglobin in stored blood, pro-inflammatory immunomodulation, endothelial dysfunction, and increased blood viscosity [31,33]. A study involving 1.29 million patients undergoing total joint arthroplasty demonstrated that anemia alone does not increase the risk of thromboembolism. In contrast, anemia necessitating blood transfusion was associated with an 83% increase in the risk of pulmonary embolism [34]. These findings indicate that transfusion itself, rather than the underlying blood loss, constitutes the primary driver of thromboembolic risk.

In our study, a notable discrepancy was observed in TXA administration rates based on fracture type: although utilization was relatively higher in cases of femoral neck fractures, it was markedly lower in the intertrochanteric and subtrochanteric subgroups. The more frequent use of TXA in femoral neck fractures may be attributed to well-established treatment standards in elective arthroplasty, where the efficacy of TXA is longer-supported by the extensive literature [12,13,30,35].

One possible explanation for the slightly higher reoperation rate in the tranexamic acid group (4.3% vs. 3.0%) is the relatively greater proportion of patients treated with total hip arthroplasty in this group (23.0% vs. 8.7%). In patients with femoral neck fractures, total hip arthroplasty is known to be associated with a somewhat higher risk of complications and subsequent reoperation compared with hemiarthroplasty [36]. This could explain the correlation. Unfortunately, we cannot draw any more specific conclusions from the data.

Regarding the timing of administration, our subgroup analysis failed to demonstrate any additional benefit for a dual-dose regimen (pre- and intraoperative) over a single intraoperative dose in terms of transfusion avoidance. In our subgroup analysis, the repeated administration of TXA was associated with a slightly heightened incidence of thromboembolic events. However, given the small number of patients in both groups, this finding should be interpreted with caution and requires further research. To date, studies investigating the efficacy and safety, including associated complications, of repeated TXA doses compared with a single administration are limited in number, and their findings are conflicting, precluding a clear clinical consensus. Narkbunnam et al. demonstrated in a randomized controlled trial that a two-dose regimen of TXA (750 mg administered preoperatively and an additional 750 mg 3 h later) resulted in significantly better outcomes than a standard single-bolus regimen [37]. Although the incidence of thromboembolic events was slightly higher in the two-dose group, this difference did not reach statistical significance. Conversely, a meta-analysis by Masouros et al. found no significant difference in the reduction in total blood loss between single-dose and multiple-dose regimens; likewise, no significant difference was observed in the incidence of thromboembolic complications [38]. Methodological constraints may explain the lack of additional benefit observed with repeated TXA dosing. Our data indicate that multi-dose protocols were preferentially utilized in femoral neck fractures treated with endoprostheses, whereas subtrochanteric and pertrochanteric fractures remained underrepresented in this subgroup. Because the latter group represents the population with the greatest potential for blood loss reduction, this distributional disparity constitutes a significant confounder in evaluating the efficacy of multi-dose vs. single-dose administration [39,40,41].

While the scale and precision of our dataset provide significant statistical power, the study’s registry-based design presents limitations. The choice of surgical procedure and prior anticoagulant therapy were not included in the matching criteria. Consequently, an imbalance was observed between the groups, with a slightly higher proportion of patients receiving anticoagulant therapy in the tranexamic acid group (52.8% vs. 50.31%; *p* = 0.037). In addition, total hip arthroplasty was performed more frequently in the tranexamic acid group (23.2% vs. 8.0%). Although sensitivity analyses incorporating both parameters (surgical procedure and prior anticoagulation therapy) revealed no shift in the effect estimates, it should be noted that both factors may be associated with increased perioperative blood loss and should therefore be considered potential confounders. The voluntary 120-day follow-up and the subsequent limited completion rate raise concerns regarding non-response bias, particularly among the most vulnerable patients. This may have resulted in an incomplete capture of long-term thromboembolic events. Furthermore, the registry’s scope is primarily surgical; the absence of data on internal and neurological complications limits our ability to accurately evaluate the efficacy of tranexamic acid administration. Furthermore, the dataset lacks granular information regarding the specific dosage and the precise timing of tranexamic acid administration.

## 5. Conclusions

This study demonstrates a strong association between the intraoperative administration of tranexamic acid (TXA) and reduced blood loss and transfusion requirements in geriatric patients undergoing surgery for proximal femoral fractures. The observed clinical benefit appeared to be consistent across fracture morphologies, and degrees of frailty, without evidence of an associated increase in thromboembolic complications. Consequently, TXA may be considered as a valuable component of perioperative care in this vulnerable patient population. Further investigation into optimized dosing schedules, including early pre-hospital or emergency room administration, is required. Future studies should utilize large cohorts to ensure adequate power for subgroup analyses based on fracture classification, thereby refining the clinical indications for multi-dose TXA therapy.

## Figures and Tables

**Table 1:** Preoperative patient data before and after matching.

	TXA *n* = 4176 (Before Matching)	No TXA *n* = 23,802 (Before Matching)	TXA *n* = 3697 (After Matching)	No TXA *n* = 3697 (After Matching)
**Age at admission [Years]**				
Min/max	70/102	70/108	70/102	70/104
Mean (standard deviation)	83.95 (6.34)	84.28 (6.57)	83.87 (6.35)	83.88 (6.28)
**Sex % (*n*)**				
Male	30.46 (1271)	29.52 (6909)	30.51% (1128)	30.43% (1125)
Female	69.54 (2902)	70.48 (16,497)	69.49% (2569)	69.57% (2572)
**ASA-Score % (*n*)**				
Median [IQR]	3 [2, 3]	3 [3, 3]	3 [2, 3]	3 [2, 3]
ASA 1	0.89 (36)	0.94 (213)	0.87% (32)	0.87% (32)
ASA 2	25.11 (1017)	22.66 (5145)	25.10% (928)	25.05% (926)
ASA 3	67.73 (2743)	69.77 (15,844)	67.87% (2509)	67.92% (2511)
ASA 4	6.20 (251)	6.57 (1493)	6.09% (225)	6.09% (225)
ASA 5	0.89 (36)	0.06 (13)	0.08% (3)	0.08% (3)
**Walking ability before fracture % (*n*)**				
Able to walk independently or with help	97.81 (3835)	97.25 (20,854)	97.75% (3614)	97.75% (3614)
Not able to walk	2.19 (86)	2.75 (590)	2.25% (83)	2.25% (83)
**Fracture type % (*n*)**				
Femoral neck	68.67 (2862)	40.81 (9535)	68.84% (2545)	68.84%% (2545)
Pertrochanteric	16.87 (703)	48.74 (11,389)	16.61% (614)	16.61% (614)
Subtrochanteric	2.88 (120)	3.81 (890)	2.60% (96)	2.60% (96)
Periprosthetic	9.62 (401)	4.07 (950)	9.98% (369)	9.98% (369)
Peri-implant	1.18 (49)	0.63 (147)	1.16% (43)	1.16% (43)
Others	0.79 (33)	1.94 (454)	0.81% (30)	0.81 (30)

* *p* < 0.05.

**Table 2:** Anticoagulation status at admission.

	TXA *n* = 3629	No TXA *n* = 3572
Anticoagulation at admission % (*n*) *		
yes	52.80% (1916)	50.31% (1797)
no	47.20% (1713)	49.69% (1775)
Type of anticoagulation % (*n*) ***		
Acetylsalicylic acid	24.01% (872)	24.4% (871)
Vitamin K antagonist	3.1% (114)	2.5% (90)
Direct factor Xa inhibitor	22.2% (806)	20.4% (727)
Direct thrombin inhibitors	0.7% (26)	1.0% (36)
Heparin/heparinoids others	2.0% (73)	2.4 (85)
Other antiplatelet agents	1.9% (68)	1.1 (38)
Other anticoagulants	0.4 (14)	0.5 (18)

* *p* < 0.05, *** multiple answers possible.

**Table 3:** Additional intraoperative anticoagulation.

	TXA	No TXA
Additional intraoperative anticoagulation therapy % (*n*) *		
Fibrinogen (*n* = 6882, missings= 512) *		
Yes	0.82 (26)	0.05 (2)
No	99.18 (3160)	99.95 (3694)
PCC (*n* = 6882, missings = 512) *		
Yes	1.47 (47)	0.43 (16)
No	98.53 (3140)	99.57 (3679)
**Vitamin K (*n* = 6885, missings = 509)**		
Yes	0.13 (4)	0.14 (5)
No	99.87 (3186)	99.86 (3690)
Antidot DOA (*n* = 6879, missings = 515) *		
Yes	0.50 (16)	0.14
No	99.50 (3167)	99.86 (3691)

* *p* < 0.05.

**Table 4:** Inpatient and 120-day follow-up data.

	TXA	No TXA
**Died (inpatient stay) % (*n* = 7382)** OR 1.0 (95%CI 0.81–1.2)		
Yes	5.34 (156)	5.09 (188)
No	95.69 (3461)	96.96 (3477)
**Died during (120-day follow-up) % (*n* = 1974)** OR 0.72 (95%CI 0.35–1.5)		
Yes	9.57 (99)	9.69 (91)
No	90.43 (936)	90.31(848)
Reoperation (inpatient stay) % (*n* = 7203) * OR 1.4 (95%CI 1.1–1.8)		
Yes	4.31 (156)	3.04 (109)
No	95.69 (3461)	96.96 (3477)
**Reoperation (120-day follow-up) % (*n* = 1746)** OR 0.75 (95%CI 0.26–2.2)		
Yes	3.15 (29)	4.00 (33)
No	96.85 (892)	96.00 (792)
**Non-surgical complications (inpatient stay) % (*n* = 7228)** OR 1.1 (95%CI 0.96–1.2)		
Yes	39.96 (1447))	38.84 (1401)
No	60.04 (2174)	61.16 (2206)
**Non-surgical complications (120 days post op) % (*n* = 1684)** OR 1.8 (95%CI 0.79–4.0))		
Yes	10.42 (92)	7.99 (64)
No	89.58 (791)	92.01 (737)
**Thromboembolic complications (inpatient stay) % (*n* = 7394)** OR 1.2 (95%CI 0.82–1.7)		
Yes	1.70 (63)	1.87 (69)
No	98.30 (3634)	98.13 (3628)
**Thromboembolic complications (120 days post op) % (*n* = 1974)** OR 2.0 (95%CI 0.18–22)		
Yes	0.39 (4)	0.96 (9)
No	99.61 (1031)	99.04 (930)

* *p* < 0.05.

**Table 5:** Hemostatic management.

	TXA	No TXA
Red blood cell units (*n* = 4106) * OR 0.82 (95%CI 0.69–0.98)		
At least one unit	44.64 (996)	52.16 (978)
No	55.36 (1235)	47.84 (897)
Fresh frozen plasma % (*n* = 3073) * OR 2.5 (95%CI 1.3–4.8)		
At least one unit	4.66 (81)	2.63 (35)
No	95.34 (1659)	97.37 (1298)
**Platelet concentrate % (*n* = 3025)** OR 1.4 (95%CI 0.55–3.4)		
At least on unit	1.47 (25)	1.13 (15)
No	98.53 (1675)	97.37 (1310)

* *p* < 0.05.

**Table 6:** Patient data subgroups after matching.

	2x TXA *n* = 225	TXA *n* = 450
**Age at admission**		
Min/max	70/96	70/97
Mean (standard deviation)	83.07 (6.04)	82.97 (5.88)
**Sex % (*n*)**		
male	29.78 (67)	29.56 (133)
female	70.22 (158)	70.44 (317)
**ASA-Score % (*n*)**		
ASA 1	2.67(6)	2.67 (12)
ASA 2	34.67(78)	34.44 (155)
ASA 3	60.00 (135)	60.22 (271)
ASA 4	2.67 (6)	2.67 (12)
**Walking ability before fracture % (*n*)**		
Able to walk independently or with help	98.67 (219)	98.67% (444)
Not able to walk	1.33 (3)	1.33 (6)
**Fracture type % (*n*)**		
Femoral neck	80.44 (181)	80.44 (362)
Pertrochanteric	8.89 (20)	8.89 (40)
Subtrochanteric	0.89 (2)	0.89 (4)
Periprosthetic	8 (18)	8 (36)
Peri-implant	1.33 (3)	1.33 (6)
Others	0.44 (1)	0.44 (2)
**Surgical procedure % (*n*)**		
Proximal femur nail	9.3 (21)	10.2 (46)
Dynamic hip screw/canulated screws	0	1.5 (7)
Total hip replacement	19.1 (43)	46.0 (207)
Hemiprothesis	60.4 (136)	33.1 (149)
Others	4.4 (10)	2 (9)
**Anticoagulation at admission % (*n*)**		
Yes	42.79 (95)	46.82 (206)
No	57.21 (127)	53.18 (234)

* *p* < 0.05.

**Table 7:** Results subgroup analysis.

	2xTXA	TXA
**Died (inpatient stay) % (*n* = 673)** OR 0.93 (95%CI 0.44–1.88)		
Yes	4.89 (11)	4.24 (19)
No	95.11 (214)	95.76 (429)
**Died during (120-day follow-up) % (*n* = 153)** OR 2.25 (95%CI 0.74–6.46)		
Yes	13.95 (6)	9.09 (10)
No	86.05 (37)	90.91 (100)
**Reoperation (inpatient stay) % (*n* = 663)** OR 1.07 (95%CI 0.46–2.35)		
Yes	4.02 (9)	4.10 (18)
No	95.98 (215)	95.90 (421)
**Reoperation (120-day follow-up) % (*n* = 135)** OR 3.37 (95%CI 0.5–22.52)		
Yes	5.41 (2)	3.06 (3)
No	94.59 (35)	96.94 (95)
**Non-surgical complications (inpatient stay) % *(n* = 661)** OR 0.99 (95%CI 0.71–1.38)		
Yes	37.56 (83)	30.91 (136)
No	62.44 (138)	69.09 (304)
**Non-surgical complications (120 days post op) % (*n* = 121)** OR 1.19 (95%CI 0.29–3.83)		
Yes	11.54 (3)	6.32 (6)
No	88.46 (23)	93.68 (89)
Thromboembolic complications (inpatient stay) % (*n* = 675) * OR 2.67 (95%CI 0.95–7.86)		
Yes	3.56 (8)	1.11 (5)
No	96.44 (217)	98.89 (445)
**Thromboembolic complications (120 days post op) % (*n* = 153)** -		
Yes	0	0
No	100 (43)	100 (110)
**Red blood cell units (*n* = 431)** OR 0.67 (95%CI 0.42–1.02)		
At least one unit	34.39 (54)	34.67 (95)
No	65.61 (103)	65.33 (179)
**Fresh frozen plasma % (*n* = 354)** OR 0.46 (95%CI 0.21–1.04)		
At least one unit	1.52 (2)	2.25 (5)
No	98.48 (130)	97.75 (217)
**Platelet concentrate % (*n* = 351)** OR 1.44 (95% CI 0.3–6.9)		
At least one unit	2.29 (3)	0.91 (2)
No	97.71 (128)	99.09 (218)

* *p* < 0.05.

## Data Availability

The original contributions presented in this study are included in the article. Further inquiries can be directed to the corresponding author.
